# A Mixture of Cod and Scallop Protein Reduces Adiposity and Improves Glucose Tolerance in High-Fat Fed Male C57BL/6J Mice

**DOI:** 10.1371/journal.pone.0112859

**Published:** 2014-11-12

**Authors:** Hanne Sørup Tastesen, Alexander Krokedal Rønnevik, Kamil Borkowski, Lise Madsen, Karsten Kristiansen, Bjørn Liaset

**Affiliations:** 1 Department of Biology, University of Copenhagen, Copenhagen, Denmark; 2 National Institute of Nutrition and Seafood Research, Bergen, Norway; Monash University, Australia

## Abstract

Low-protein and high-protein diets regulate energy metabolism in animals and humans. To evaluate whether different dietary protein sources modulate energy balance when ingested at average levels obesity-prone male C57BL/6J mice were pair-fed high-fat diets (67 energy percent fat, 18 energy percent sucrose and 15 energy percent protein) with either casein, chicken filet or a mixture of cod and scallop (1∶1 on amino acid content) as protein sources. At equal energy intake, casein and cod/scallop fed mice had lower feed efficiency than chicken fed mice, which translated into reduced adipose tissue masses after seven weeks of feeding. Chicken fed mice had elevated hepatic triglyceride relative to casein and cod/scallop fed mice and elevated 4 h fasted plasma cholesterol concentrations compared to low-fat and casein fed mice. In casein fed mice the reduced adiposity was likely related to the observed three percent lower apparent fat digestibility compared to low-fat, chicken and cod/scallop fed mice. After six weeks of feeding an oral glucose tolerance test revealed that despite their lean phenotype, casein fed mice had reduced glucose tolerance compared to low-fat, chicken and cod/scallop fed mice. In a separate set of mice, effects on metabolism were evaluated by indirect calorimetry before onset of diet-induced obesity. Spontaneous locomotor activity decreased in casein and chicken fed mice when shifting from low-fat to high-fat diets, but cod/scallop feeding tended (*P* = 0.06) to attenuate this decrease. Moreover, at this shift, energy expenditure decreased in all groups, but was decreased to a greater extent in casein fed than in cod/scallop fed mice, indicating that protein sources regulated energy expenditure differently. In conclusion, protein from different sources modulates energy balance in C57BL/6J mice when given at normal levels. Ingestion of a cod/scallop-mixture prevented diet-induced obesity compared to intake of chicken filet and preserved glucose tolerance compared to casein intake.

## Introduction

Identifying nutritional strategies to alleviate the obesity pandemic are of great interest. Diet-induced thermogenesis, i.e. the regulated liberation of energy in the form of heat [1], could lower food efficiency, and thereby diminish obesity development. Already in 1939, induction of adaptive thermogenesis by feeding rats very low (4–8 weight percent) or very high (54 weight percent) protein diets was described [2]. Later, the increment in thermogenesis by low-protein (LP) diets was verified in rats [3], [4], in baby pigs [5], and similar effects were observed in young human subjects [6]. Thus, intake of LP diets induces thermogenesis, but instead of resulting in decreased body mass, the reduced food efficiency is compensated for by a higher food intake [7].

Whereas LP diets may increase energy intake, high-protein (HP) diets are more satiating than an isoenergetic amount of carbohydrates or fat [8], [9]. Moreover, HP intake induces higher postprandial thermogenesis than high-carbohydrate ingestion does [10], [11]. It is likely that both reduced energy intake and elevated thermogenesis might be underlying mechanisms explaining, at least in part, the reduction in body mass observed in mice [12]–[15] and humans [9], [16], [17] by replacing carbohydrates with protein.

Despite the known effects of LP and HP diets on thermogenesis, limited information exists on whether varying protein sources affect body mass and composition differently [18]. From studies in rodents, we know that consumption of hydrolyzed rather than intact proteins reduces body mass gain, adipose tissue mass and hepatic and plasma lipid concentrations [19]–[21]. Moreover, whey ingestion decreased fat mass relative to casein intake in mice [22]–[24], and an intervention study with free-living overweight and obese subjects indicated that intake of whey protein, but not soy protein (both approximately 56 g protein/day for 23 weeks) resulted in a significant reduction in body mass, fat mass and waist circumference, relative to the carbohydrate (maltodextrin) control treatment [25]. In a randomized, double-blinded intervention study with cross-over design, ingestion of a liquid test meal consisting of 50% whey protein, 40% carbohydrate and 10% fat, induced a higher postprandial thermic effect than equal amounts of casein and soy protein [26]. Thus, studies in both rodents and humans indicate that different protein sources might affect body weight gain and adiposity differently.

The average (hereafter referred to as normal) protein intake in humans has been estimated to be 15–16 energy percent (E%) both in the US [27] and in the UK [28]. We have recently shown that obesity-prone C57BL/6J mice exhibited distinct metabolic responses to intake of various dietary protein sources, given as 15 E% protein in a high-fat (HF) background-diet [29]. Mice fed scallop muscle as the sole protein source were protected against diet-induced obesity, enlarged liver mass and hyperlipidemia as compared to mice fed chicken or cod filets. However, the scallop fed mice also had lower *ad libitum* feed intake, suggesting different satiating effects of the protein sources [29]. Therefore, the present study was undertaken in order to elucidate whether the protein sources casein (a commonly used standard reference protein), chicken breast filet or a mixture of cod filet and scallop muscle, would affect diet-induced obesity during equal energy intake (pair-feeding) in HF diets fed to male C57BL/6J mice for seven weeks. Furthermore, to evaluate instant differences in metabolism independent of the development of obesity indirect calorimetry was performed during the first 72 h of feeding on the HF diets containing protein from different sources. Protein from different sources at normal level was found to modulate energy balance in C57BL/6J mice, and consumption of a cod/scallop-mixture prevented HF diet-induced development of obesity compared to chicken and preserved glucose tolerance compared to casein.

## Materials and Methods

### Ethics Statement

The animal experiments were approved by the Norwegian National Animal Health Authorities (Experiment 1 (Expt. 1) performed in Norway, permit number 3421) and the Danish National Animal Experiments Inspectorate (Experiment 2 (Expt. 2) performed in Denmark, permit number 2012-15-2934), and care and handling were carried out in strict compliance with the ethical standards of the 1964 Helsinki Declaration, as revised in 1983. No adverse events were observed. To ameliorate suffering the mice were anaesthetized by inhalation of isoflurane (4%, Isoba Vet) before being euthanized by exsanguination by cardiac puncture.

### Experimental diets

Low-fat (LF) diet (10 E% fat, 70 E% carbohydrate and 20 E% protein, OpenSource Diet no. D12450B, Research Diets, NJ, USA) was used to feed mice during acclimatization periods and as a reference diet. Three isoenergetic experimental HF diets (67 E% fat, 18 E% carbohydrate (sucrose) and 15 E% protein) were made with protein from different sources; casein sodium salt from bovine milk (casein, Sigma C-8456) chicken breast filets (chicken) and a mixture of wild caught cod filets and Canadian scallop muscles (cod/scallop, 1∶1 on amino acid content) as previously described [29], [30] with the modification that three g cystine/kg diet were added to all diets in the present study. Raw chicken breast filets, raw cod filets and raw scallop muscle were minced, freeze dried and powdered before being added into the diets and apart from the supplemented cystine, the protein sources casein, chicken filet or cod filet and scallop represented the sole amino acid source in the respective diets. The final compositions of the diets are shown in Table 1 and Table 2.

**Table 1:** Composition of the experimental diets.

	LF^a^	Casein^b^	Chicken^c^	Cod/scallop^d^
*Composition (g/kg)*				
Casein	190	215	−	−
Chicken	−	−	240	−
Cod	−	−	−	114
Scallop	−	−	−	133
L-Cystine	2.8	3	3	3
KCl	−	10.2	5.4	−
Corn starch	299	−	−	−
Maltodextrin 10	33	−	−	−
Sucrose	332	242	223	221
Lard	19	198	198	198
Vegetable oil^e^	23.7	198	198	198
Cellulose	47.4	50	50	50
AIN-76 mineral mix	9.5^f^	67	67	67
AIN-76 vitamin mix	9.5^g^	14	14	14
Choline bitartrate	1.9	2	2	2
Butylated hydroxytoluene	−	0.4	0.4	0.4
DiCalcium Phosphate	12.3	−	−	−
Calcium Carbonate	5.2	−	−	−
Potassium Citrate, 1 H_2_O	15.6	−	−	−
*Analyzed (g/kg)*				
Crude protein^h^	170	190	190	200
Ash	31	48	60	75
Fat	44	390	400	390
Gross energy kJ/g	17.4	26.2	26.0	25.5

a OpenSource diet no. D12450B (Research Diets, Inc. NJ, USA).

b Casein (cat. no. C8654, lot BCBC 3986, Sigma-Aldrich, MO, USA).

c Chicken breast fillets (Kyllingfilet naturell, Ytterøykylling AS, Norway).

d Cod fillets (Wildcaught in the Northeastern Atlantic) and Canadian scallops, (Wild North Atlantic scallops, 20–30 ct, Placopecten magellanicus, Clearwater Seafoods Limited, NS, Canada).

e LF: soybean oil. Casein, chicken and cod/scallop: corn oil.

f Mineral Mix S10026.

g Vitamin Mix V100001.

h Crude protein, N ^x^ 6.15 for casein; N ^x^ 5.6 for chicken filet and cod/scallop.

**Table 2:** Amino acid composition of the experimental diets.

	LF	Casein	Chicken	Cod/scallop
*mmol/kg*				
Ala	60	68	138	119
Arg	31	36	66	73
Asx^a^	100	109	154	149
Cys	28	36	48	42
Glx^b^	274	307	212	191
Gly	40	47	109	192
His*	29	34	43	21
Ile*	66	77	75	61
Leu*	120	142	126	107
Lys*	99	112	136	116
Met*	29	34	36	34
Phe*	51	59	48	41
Pro	153	185	61	47
Ser	94	107	78	73
Thr*	63	70	78	64
Trp	9	11	11	8
Tyr	36	44	28	24
Val*	94	111	88	69
Hyp	<0.1	<0.1	3	3
Tau	<0.1	<0.1	1	61
EAA^c^	551	640	630	512
BCAA^d^	280	330	290	237
Total AA^e^	1376	1590	1540	1494

* essential animo acids.

a Asx: sum of Asp + Asn.

b Glx: sum of Glu + Gln.

c EAA: sum of essential amino acids.

d BCAA: sum of branched-chain amino acids.

e Total AA: total sum of amino acids.

### Animal studies

Male C57BL/6JBomTac mice (Taconic, Ejby, Denmark) 8 to 9 weeks old and weighing approximately 25 g at arrival were housed individually at thermoneutrality (28±1°) under a 12 h light-dark cycle. The mice were fed LF diet during acclimatization to the animal facility. Two experiments were carried out as follows; Expt. 1 encompassed 32 mice (*n* = 8/group) which were assigned into four experimental groups by bodyweight after five days acclimatization and fed either LF diet or HF diets (Table 1 and 2) for seven weeks. At week six the mice were subjected to an oral glucose tolerance test (O-GTT). After seven weeks the mice were terminated and the following tissues were dissected out, weighed and frozen at −80°C; liver, kidneys, heart, skeletal muscle soleus, epididymal white adipose tissue (eWAT), perirenal/retroperitoneal white adipose tissue (p/rWAT), inguinal white adipose tissue (iWAT), and interscapular brown adipose tissue (iBAT). Feed efficiency was calculated as body mass gain per energy intake (g BM/MJoule), based on the data from week six, i.e., prior to the O-GTT. Expt. 2 encompassed 30 mice (*n* = 10/group). After seven days acclimatization on LF diet the mice were placed in indirect calorimetry cages for 72 hours of baseline indirect calorimetry and activity measurements while still on LF diet. Based on body weight and baseline measurements of total activity and RER in light and dark phases the mice were divided into three groups and fed the experimental HF diets for another 72 hours of measurements and subsequently terminated. The mice were in both experiments anaesthetized by inhalation of isoflurane (4%, Isoba Vet) and euthanized by exsanguination by cardiac puncture after a 4 h fast. The blood was heparinized (20.2 units sodium heparin/mL blood), centrifuged (4°C, 2500 g, 5 min) and plasma fractions were stored at −80°C until analysis. To ensure comparability between Expt. 1 and Expt. 2. handling and care of the mice were standardized between the two experiments and mice from the same supplier and feed from the same batch were used for both experiments.

### Pair-feeding

LF fed mice had free access to feed, whereas HF fed mice were pair-fed for the duration of both Expt. 1 and Expt. 2 to obtain equal energy intake. In Expt. 1 leftover diet was collected from every cage three times per week and intake calculated accordingly. On a group basis casein fed mice had slightly lower feed intake than chicken and cod/scallop fed mice and as a consequence hereof the mice in the chicken and cod/scallop fed groups were mildly feed restricted compared to the casein fed mice to obtain similar energy intakes between groups. The average daily food intake in grams for Expt. 1 was 3.01 g/d in LF fed mice, 2.06 g/d in casein fed mice, 2.20 g/d in chicken fed mice and 2.31 g/d in cod/scallop fed mice. The daily energy intake was 52.4 kJ/d in LF fed mice, 53.9 kJ/d in casein fed mice, 57.2 kJ/d (+6.0% vs. casein) in chicken fed mice and 58.8 kJ/d (+9.1% vs. casein) in cod/scallop fed mice. The pair-feeding during Expt. 2 was essentially done in the same way, however leftovers were collected and the mice fed every day to keep the energy intake similar between groups during this shorter study.

### Diet composition analyses

Diets were analyzed as previously described [29]. In short; energy contents were determined by bomb calorimetry (Parr Instruments, Moline, IL, USA). For total amino acid analysis norvaline was added to samples as internal standard, samples were hydrolyzed (6 M HCl, 110±2°C, 22 h) and derivatized (AccQ-Tag Ultra Derivatization Kit, Waters, MA, USA). Amino acids were separated and detected on the ACQUITY UPLC System (Waters, MA, USA), identified using Pierce Amino Acid Standard H (Thermo Fisher Scientific Inc., IL, USA) to which norvaline, taurine and hydroxy-proline were added and finally quantified by internal and external standard regression. For tryptophan analysis the samples were hydrolyzed (Ba(OH)_2_,110±2°C, 20 h), pH adjusted to 6.2, separated by HPLC (Shimadzu 6A/6B) equipped with a SUPELCOSILTM LC-18 HPLC-column, detected in UV-spectrophotometer (Shimadzu SPD 6A) at 280 nm and quantified using a standard curve of L-Tryptophan (T-0254, Sigma-Aldrich). Total cysteine was determined, at the Norwegian Institute of Food, Fishery and Aquaculture, after oxidation of cysteine/cystine (9∶1 performic acid (88%): H_2_O_2_ (30%) (v/v)) to yield cysteic acid.

### Feces collection

After six weeks of feeding (Expt. 1) the mice were placed in cages with the standard wood chip layer replaced by paper lining for the purpose of collecting feces for one week. Feces left behind in cages were collected, weighted and frozen at −80°C until analyses for nitrogen and total fat content. Based on feces measurements and diet-intake data apparent digestibility of fat and nitrogen was calculated as follows: 100× (intake (mg) - fecal output (mg))/(intake (mg)).

### Nitrogen and fat content in diets and feces

Nitrogen content was determined by the Dumas method using Leco FP-528 nitrogen analyzer (Leco Corp, MI, USA). The crude protein content in the diets was calculated as nitrogen content multiplied by 6.15 for casein and 5.6 for chicken filet and cod/scallop [31]. Total fat content was determined gravimetrically after extraction with organic solvents before and after acidic hydrolysis as described previously [29].

### Plasma measurements

MaxMat PL II analyzer (MAXMAT S.A., Montpellier, France) and conventional kits were used to measure 4 h fasted plasma lactate (Sentinel Diagnostics, Italy), triglyceride (TG), total cholesterol, LDL cholesterol, glucose and alanine aminotransferase (MaxMat, France), hydroxyl-butyrate (OH-butyrate) and glycerol (Randox, UK), free fatty acids (FFA), HDL cholesterol and total bile acids (Dialab, Austria) concentrations. 4 h fasted plasma insulin concentrations were analyzed using DRG mouse insulin ELISA kit (DRG Diagnostics, Germany).

### Liver lipid analysis

Total liver lipids were extracted with chloroform:methanol (2∶1, v∶v). Lipid classes were analyzed via automated Camaq HPTLC system and separated on HPTLC silica gel 60 F plates as previously described [32].

### qRT-PCR

Gene expression analysis was performed as described previously [21]. In short, total RNA was isolated from tissue samples with TRIzol Reagent (Invitrogen, Thermo Fisher Scientific, Carlsbad, CA, USA). Qualities and concentrations of the purified RNA were assessed using NanoDrop ND-1000 UV-Vis spectrophotometer (NanoDrop Technologies, Wilmington, DE, USA). Using GeneAmp PCR 9700 (Applied Biosystems, Thermo Fisher Scientific, Carlsbad, CA, USA), TaqMan RT buffer, dNTP, oligo(dT)primers, RNase inhibitor, Multiscribe Reverse Transcriptase (N808-0234, Applied Biosystems) and RNase-free water RT reactions were performed for 60 min at 48°C. The produced cDNA was subject to qRT-PCR in LightCycler 480 Real-Time PCR System (Roche Applied Sciences, Indianapolis, IN, USA) using SYBR Green Master Mix (LightCycler 480 SYBR Green master mix kit, Roche Applied Sciences) and gene-specific primers (Table S1). Data were analyzed as a ratio between gene of interest and a reference gene, TATA box binding protein (*Tbp*), and normalized to the mean of the LF samples.

### Oral glucose tolerance test

After six weeks on experimental diets mice were subjected to 6 h fasted O-GTT. Early in the morning of the test day mice were placed in cages without feed and after six hours fasted blood glucose was measured in whole blood, taken from the tail vein by a Bayer Contour glucometer and glucose test strips (Bayer, Germany). Glucose was administered by oral gavage (2 mg glucose/g body mass) and blood glucose concentration was measured 15, 30, 60 and 120 minutes after glucose administration. Blood glucose incremental area under the curve (iAUC, mmol/L/h) was calculated as AUC above baseline value, i.e., 6 h fasted blood glucose, by applying the trapezoid rule to a plot of group mean blood glucose concentration versus time of measurements [33], [34].

### HOMA-IR and QUICKI

Based on 4 h fasted plasma glucose and insulin Homeostatic Model Assessment of Insulin Resistance (HOMA-IR) was calculated as follows: Glucose (mmol/l)×insulin (µU/ml)/22.5 [35] and Quantitative Insulin Sensitivity Check Index (QUICKI) was calculated as follows: 1/(log(insulin [mU/l])+log(glucose [mg/dl])) [36].

### Indirect calorimetry and spontaneous locomotor activity

VO_2_ and VCO_2_ was measured in open-circuit indirect calorimetry cages as described previously [21]. In short, the mice were housed in CaloCages (Phenomaster, TSE Systems), equipped with infrared light-beam frames (ActiMot2). VO_2_ and VCO_2_ was measured for each cage, i.e., each mouse, for 1.9 min once every 30 min, while light-beam breaks were measured continuously. Measurements were performed for a total of 72 h while all groups were fed the LF diet and consecutively for 72 h on the respective HF diets. For each 72 h period of measurements the first 24 h were regarded as an adaptation period and only the subsequent 48 h were used for analyses; Based on two consecutive light (06.00–17.30 h) and dark (18.00–05.30 h) phases respiratory exchange ratio (RER) was calculated from VO_2_ and VCO_2_ and spontaneous locomotor activity was defined as total counts of light-beam breaks. Energy expenditure (EE) was calculated as follows; 16.3 kJ/L × L VO_2_ +4.6 kJ/L × L VCO_2_ [37].

### Statistical analyses

The data represent group means ± SEM. After homogeneity of variances was established by Levene's test the data were subjected ANOVA analyses followed by Tukey's pair-wise comparisons and group means were considered statistically different at *P*<0.05. *P* values represent the overall outcome of the ANOVA and letters denote differences identified by post hoc tests. Repeatedly measured data, i.e., growth, energy intake, O-GTT, RER, activity and EE were analyzed by repeated measurements ANOVA followed by Tukey's post hoc.

## Results

### Reduced body mass gain, feed efficiency and adiposity in casein and cod/scallop fed mice

**Figure 1:**
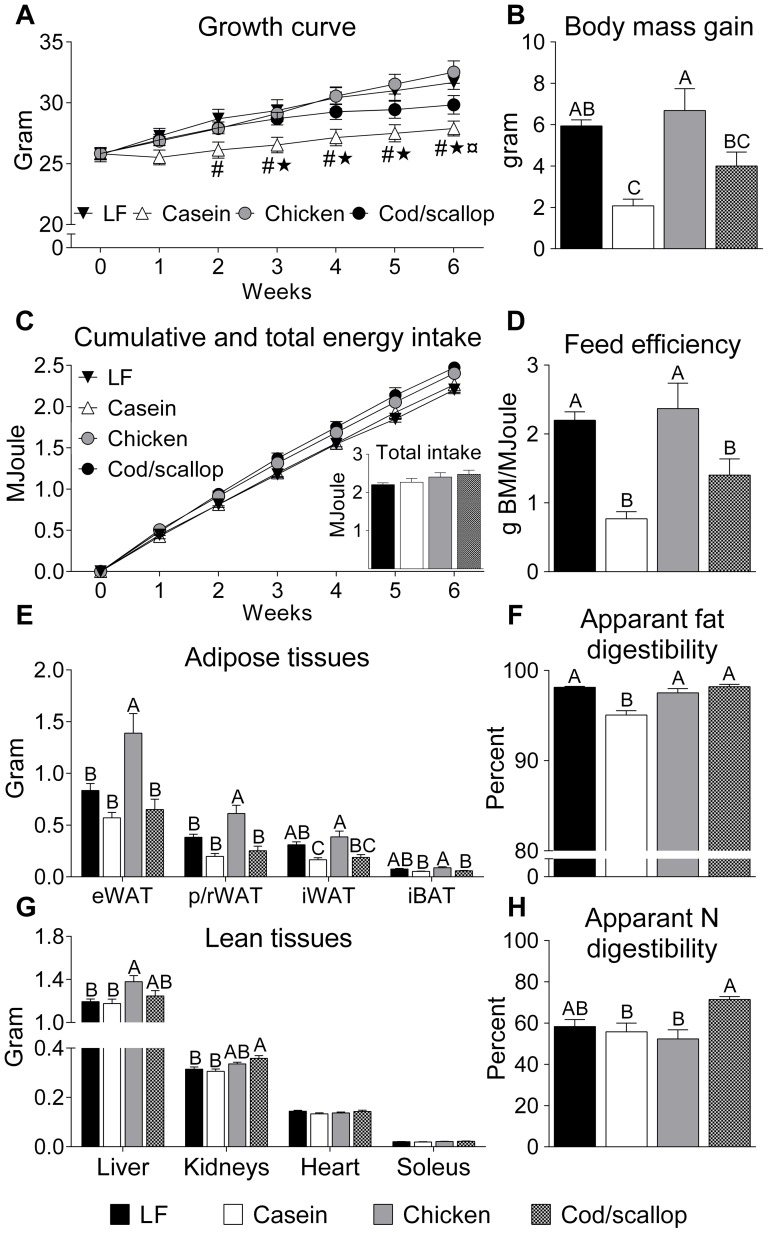
Casein and cod/scallop protein reduces body mass gain and adiposity despite equal energy intake. A: Growth curve during six weeks. B: Body mass gain. C: Cumulative and total energy intake. D: Feed efficiency. E: Adipose tissue masses. F: Apparent fat digestibility. G: Lean tissue masses. H: Apparent nitrogen digestibility. A-H in male C57BL/6J mice fed the experimental diets for six weeks. Data (Expt. 1) represent group means (*n* = 7–8) ± SEM analyzed by one-way ANOVA followed by Tukey's pair-wise comparisons. Body mass development and cumulative energy intake were analyzed by repeated measurements ANOVA followed by Tukey's post hoc. Means that do not share a letter are significantly different (*P*<0.05). ^#^ indicates significantly higher body mass in LF fed than in casein fed mice. * indicates significantly higher body mass in chicken fed than in casein fed mice. ¤ indicates significantly higher body mass in chicken fed than in cod/scallop fed mice.

Casein fed mice gained significantly less body mass than LF and chicken fed mice, whereas cod/scallop fed mice gained less weight than chicken fed mice during six weeks of feeding (Fig. 1A-B). Energy intake was not significantly different between groups (Fig. 1C) and thus the feed efficiency was lower in casein and cod/scallop fed mice compared to LF and chicken fed mice (Fig. 1D). The dietary fat intake was similar in casein fed (769±29 mg fat/24 h), chicken fed (848±34) and cod/scallop fed mice (845±37), but significantly lower in LF fed mice (144.2±2.2) than in the three HF fed groups (*P*<0.001). However, significantly more fat was excreted in the feces in the casein fed mice (38.8±4.3 mg, *P*<0.001) than in LF fed (2.67±0.11), chicken fed (20.6±3.6) and cod/scallop fed mice (14.4±1.6). Moreover, chicken and cod/scallop fed mice excreted more fat in the feces than LF fed mice. Thus, apparent fat digestibility was lower in casein fed than in LF, chicken and cod/scallop fed mice (Fig. 1F). The nitrogen intake was lower in the casein fed (61.2±2.3 mg N/24 h, *P*<0.001) than in LF fed (90.7±1.4), chicken fed (72.2±2.9) and cod/scallop fed mice (77.6±3.3). Furthermore, nitrogen intake was higher in LF fed mice compared to all three HF fed groups. The fecal excretion of nitrogen was lower in casein fed (27.1±2.9) and cod/scallop fed (22.1±1.1) than in LF fed mice (38.0±3.5). Moreover, nitrogen excretion was lower in cod/scallop fed than in chicken fed mice (33.8±2.6). Apparent nitrogen digestibility was thus higher in cod/scallop fed than in casein fed and chicken fed mice (Fig. 1H). Adiposity varied in the different groups after seven weeks of feeding (Fig. 1E); masses of eWAT and p/rWAT were lower in LF, casein and cod/scallop fed mice than in chicken fed mice. iWAT masses were lower in casein and cod/scallop fed mice than in chicken fed mice and lower in casein fed than in LF fed mice. iBAT masses were lower in casein and cod/scallop fed mice than in chicken fed mice. No differences were seen in soleus muscle and heart tissue masses between groups, but liver masses were increased in chicken fed compared to LF and casein fed mice, and kidney masses were increased in cod/scallop fed compared to LF and casein fed mice (Fig. 1G).

### Elevated plasma and liver lipids in chicken fed mice

Obesity is associated with dysregulation of plasma lipids and ectopic fat accumulation, and thus, we measured plasma and liver lipids. Plasma metabolites and liver lipids measured in the 4 h fasted state are listed in Table 3. Chicken fed mice had increased plasma total cholesterol compared to LF and casein fed mice. Furthermore, plasma HDL cholesterol and LDL cholesterol levels were increased in chicken fed compared to LF and casein fed mice and increased in cod/scallop fed compared to LF fed mice. Casein fed mice had an increased HDL:total cholesterol ratio compared to LF, chicken and cod/scallop fed mice. Total bile acid levels were increased in LF fed compared to chicken fed mice. TG concentrations were increased in LF fed compared to casein and cod/scallop fed mice, and increased in chicken fed compared to casein fed mice. Glycerol was increased in LF fed compared to casein and cod/scallop fed mice. A tendency towards increased FFA was seen in LF fed mice (*P* = 0.079). No differences were seen in 4 h fasted plasma β-hydroxybutyrate or alanine aminotransferase between the groups (Table 3). Liver TG and total neutral lipid concentrations were higher in chicken fed than in casein and cod/scallop fed mice, while free cholesterol was increased in LF fed compared to casein fed mice and steryl ester was increased in LF fed compared to casein, chicken and cod/scallop fed mice. No differences were seen in hepatic diglyceride levels between groups (Table 3).

**Table 3:** 4 h fasted plasma metabolites, liver lipids and liver relative gene expression.

	LF	Casein	Chicken	Cod/scallop	*P* value
*Plasma metabolites*					
Total cholesterol (mmol/L)	3.24±0.18^b^	3.54±0.18^b^	4.53±0.19^a^	3.88±0.15^ab^	<0.001
HDL cholesterol (mmol/L)	2.68±0.13^c^	3.14±0.13^bc^	3.72±0.17^a^	3.23±0.13^ab^	<0.001
HDL:total cholesterol ratio	0.83±0.01^b^	0.89±0.01^a^	0.82±0.01^b^	0.83±0.01^b^	0.001
LDL cholesterol (mmol/L)	0.87±0.05^c^	0.97±0.08^bc^	1.42±0.08^a^	1.16±0.07^ab^	<0.001
Total bile acids (mmol/L)	3.0±0.29^a^	2.7±0.23^ab^	1.9±0.17^b^	2.4±0.19^ab^	0.009
TG (mmol/L)	0.75±0.07^a^	0.40±0.03^c^	0.65±0.05^ab^	0.50±0.06^bc^	<0.001
FFA (mmol/L)	0.46±0.04	0.32±0.04	0.27±0.03	0.33±0.07	0.079
Glycerol (mmol/L)	0.32±0.02^a^	0.24±0.02^b^	0.26±0.02^ab^	0.25±0.02^b^	0.025
β-hydroxybutyrate (mmol/L)	0.42±0.11	0.34±0.04	0.21±0.08	0.25±0.05	0.21
Alanine aminotransferase (U/L)	24±3	28±9	26±2	50±17	0.25
*Liver lipids*					
TG (mg/g)	29±7^ab^	26±3^b^	49±9^a^	25±3^b^	0.020
Total neutral lipid (mg/g)	35±7^ab^	30±3^b^	53±9^a^	29±3^b^	0.026
Cholesterol (mg/g)	2.9±0.09^a^	2.4±0.09^b^	2.5±0.06^ab^	2.7±0.15^ab^	0.037
Steryl ester (mg/g)	3.0±0.44^a^	1.6±0.16^b^	1.6±0.19^b^	1.2±0.10^b^	<0.001
Diglycerides (mg/g)	0.2±0.06	0.2±0.03	0.2±0.03	0.1±0.02	0.52
*Liver relative mRNA expression*					
*Srebf1*	1±0.28	0.82±0.27	1.22±0.25	1.52±0.34	0.36
*Acaca*	1±0.26	0.94±0.13	1.11±0.12	1.04±0.13	0.90
*Fasn*	1±0.39	0.81±0.14	0.76±0.10	0.89±0.15	0.88
*Scd-1*	1±0.25^a^	0.06±0.01^b^	0.03±0.01^b^	0.02±0.00^b^	<0.001
*Dgat-1*	1±0.06	0.70±0.06	0.98±0.13	0.73±0.09	0.054
*Hmgcr*	1±0.15^b^	1.87±0.44^ab^	1.95±0.34^ab^	2.64±0.49^a^	0.044
*Pck-1*	1±0.12^a^	0.32±0.05^b^	0.53±0.12^ab^	0.95±0.21^a^	0.006
*Hk2*	1±0.26^a^	0.43±0.12^b^	0.51±0.10^b^	0.48±0.04^b^	0.043
*Hk4*	1±0.19	0.81±0.06	1.21±0.25	0.91±0.11	0.42
*Pfkl*	1 ±0.13^a^	0.41±0.06^b^	0.85±0.15^ab^	0.75±0.10^ab^	0.014
*Pklr*	1 ±0.25^a^	0.55±0.08^ab^	0.71±0.13^ab^	0.41±0.05^b^	0.042

Data represent group means (*n* = 6–8) ± SEM analyzed by one-way ANOVA followed by Tukey's post hoc. Means that do not share a superscript letter are significantly different (*P*<0.05). Abbreviations: TG, triglycerides; FFA, free fatty acids; *Srebf1*, sterol regulatory element-binding transcription factor 1; *Acaca*, acetyl-coenzyme A carboxylase alpha; *Fasn*, fatty acid synthase; *Scd-1*, stearoyl-CoA desaturase-1; *Dgat-1*, diacylglycerol acyltransferase-1; *Hmgcr*, 3-hydroxy-3-methylglutaryl-coenzyme A reductase; *Pck-1*, phosphoenol pyruvate carboxykinase-1; *Hk2*, hexokinase 2; *Hk4*, hexokinase 4; *Pfkl*, phosphofructokinase, liver, B-type; *Pklr*, pyruvate kinase liver and red blood cell.

### Hepatic expression of genes involved in de novo lipogenesis and gluconeogenesis is modulated by dietary protein source

Based on the differences in plasma and liver lipids we analyzed hepatic expression of mRNA encoding genes involved in lipogenesis and gluconeogenesis (Table 3). Expression of mRNA encoding stearoyl-CoA desaturase-1 (*Scd-1*), an enzyme catalyzing the conversion of SFA to MUFA, important for targeting FFA to either incorporation into lipoproteins (VLDL) or storage as TG in lipid-droplets, was higher in LF fed compared to casein, chicken and cod/scallop fed mice. Expression of mRNA encoding 3-Hydroxy-3-metylglutaryl-CoA reductase (*Hmgcr*) was increased in cod/scallop fed compared to LF fed mice. A strong tendency towards increased expression of mRNA encoding the lipogenic gene diacylglycerol acyltransferase 1 (*Dgat1*, *P* = 0.054) was seen in the LF and chicken fed mice compared to casein and cod/scallop fed mice. Expression of mRNA encoding phosphoenol pyruvate carboxykinase-1 (*Pck-1*), the rate limiting enzyme controlling gluconeogenesis by catalyzing the formation of phosphoenolpyruvate from oxaloacetate, was higher in LF and cod/scallop fed than in casein fed mice. Expression of mRNA encoding hexokinase 2 *(Hk2)* was increased in LF fed compared to casein, chicken and cod/scallop fed mice. Expression of mRNA encoding phosphofructokinase, liver, B-type *(Pfkl)* was increased in LF fed compared to casein fed mice and expression of mRNA encoding pyruvate kinase, liver and red blood cell (*Pklr*) was increased in LF fed compared to cod/scallop fed mice. No differences in expression of mRNA encoding the genes sterol regulatory element-binding transcription factor 1 (S*rebf1*), acetyl-CoA carboxylase (*Acaca*), fatty acid synthase (*Fasn*) and hexokinase 4 (*Hk4*) were observed, but expression of mRNA encoding S*rebf1* tended to be higher in cod/scallop fed than in casein fed mice (*P* = 0.067).

### Decreased glucose tolerance in casein fed and increased insulin resistance-score in chicken fed mice

**Figure 2:**
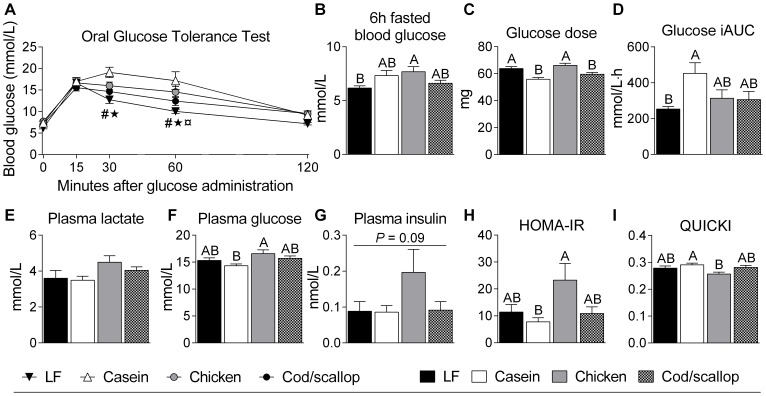
Casein tends to reduce glucose tolerance while chicken tends to cause increased plasma insulin concentration. A: Blood glucose measured before and at 15, 30, 60 and 120 minutes after oral administration of glucose (gavage, 2 mg/g body mass) during 6 h fasted oral glucose tolerance test in mice after six weeks on the experimental diets (O-GTT). B: 6 h fasted blood glucose. C: glucose dose administered by oral gavage. D: incremental blood glucose area under the curve (iAUC). E: Plasma lactate. F: Plasma glucose. G: Plasma insulin. E-G: concentrations measured in 4 h fasted plasma collected at the termination of the mice after seven weeks on the experimental diets. H: Homeostatic Model Assessment of Insulin Resistance (HOMA-IR) scores. I: Quantitative Insulin Sensitivity Check Index (QUICKI) scores. H-I: The scores were calculated based on 4 h fasted plasma glucose and insulin levels. Data (Expt. 1) represent group means (*n* = 7–8) ± SEM analyzed by one-way ANOVA followed by Tukey's pair-wise comparisons. O-GTT curve was analyzed by repeated measurements ANOVA followed by Tukey's post hoc. Means that do not share a letter are significantly different (*P*<0.05). ^#^ indicates significantly higher blood glucose in casein fed than in LF fed mice. * indicates significantly higher blood glucose in casein fed than in cod/scallop fed mice. ¤ indicates significantly higher blood glucose in chicken fed than in LF fed mice.

As obesity, visceral adiposity, and hepatic steatosis have been shown to associate with impaired glucose and insulin homeostasis, we subjected the mice to 6 h fasted O-GTT after six weeks of feeding. Chicken fed mice had increased 6 h fasted blood glucose levels compared to LF fed mice (Fig. 2A-B). Casein fed mice had higher blood glucose concentrations compared to LF and cod/scallop fed mice 30 and 60 minutes after glucose administration and chicken fed mice had higher blood glucose concentrations compared to LF fed mice 60 after administration (Fig. 2A). The glucose was administered according to body mass (2 mg glucose/g BM) and LF and chicken fed mice thus received a greater load of glucose than casein and cod/scallop fed mice (Fig. 2C). The calculated iAUC blood glucose (Fig. 2D) was higher in casein fed than in LF fed mice and furthermore tended to be higher in casein fed mice than in chicken and cod/scallop fed mice (*P* = 0.09). In 4 h fasted plasma collected at the termination of the mice after seven weeks of feeding, glucose concentrations were higher in chicken fed than in casein fed mice (Fig. 2F), while insulin concentrations tended to be increased in chicken fed mice (*P* = 0.09, Fig. 2G). HOMA-IR insulin-resistance-scores were higher in chicken fed than in casein fed animals and tended to be higher in chicken fed than in cod/scallop fed mice (*P* = 0.08, Fig. 2H). QUICKI insulin-sensitivity-scores were higher in casein fed than in chicken fed mice (Fig. 2I).

### Difference in RER between light and dark phases abolished by HF feeding

**Figure 3:**
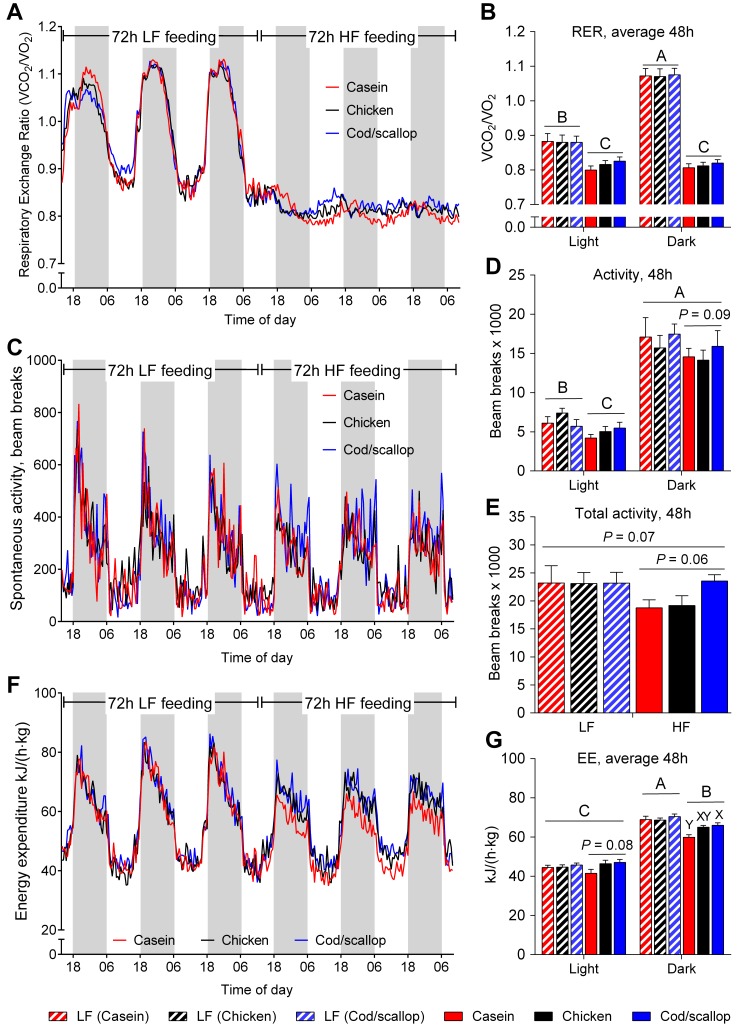
Protein sources affect energy expenditure and tend to affect spontaneous locomotor activity. A: RER in mice fed LF diet for 72 h and HF diets for 72 h in open-circuit indirect calorimetry cages. B: Average respiratory exchange ratio (RER) during 48 h on LF diet and HF diets in light and dark phases. C: Spontaneous locomotor activity during 72 h on LF diet and 72 h on HF diets. D: Spontaneous locomotor activity in light and dark phases during 48 h in mice fed LF diet and HF diets. E: Total spontaneous locomotor activity during 48 h in mice fed LF diet and HF diets. F: Energy expenditure (EE) during 72 h on LF diet and 72 h on HF diets. G: Average EE during 48 h in light and dark phases in mice fed LF diet and HF diets. Data (Expt. 2) represent group means (*n* = 9–10) ± SEM analyzed by ANOVA followed by Tukey's pair-wise comparisons. RER, activity and EE data were analyzed by repeated measurements ANOVA followed by Tukey's post hoc. Means that do not share a letter are significantly different (*P*<0.05).

To elucidate whether altered EE was an underlying mechanism behind differences in fat accretion, we utilized indirect calorimetry. During the 48 h of indirect calorimetry measurements that were analyzed, LF fed mice had higher RER in dark than in light phases (*P*<0.0001, Fig. 3A-B). After the shift to HF diets, RER decreased in both light and dark phases and the difference between light and dark phases was no longer evident (Fig. 3A-B). The different protein sources caused no differences in RER between the groups neither in light nor in dark phases (Fig. 3B).

### Increased EE and a tendency towards increased activity in cod/scallop fed mice

Similarly to RER, activity level differed between light and dark phases in LF fed mice with higher activity levels during dark phases (*P*<0.0001, Fig. 3C-D). The initial activity levels measured while the mice were fed the LF diet were similar between groups and higher in dark than in light phases (Fig. 3D-E). Changing to HF diets did not change the activity during the dark phases (Fig 3D). However, HF feeding decreased activity level during light phases (*P* = 0.018, Fig. 3D). Total activity tended to decrease with the shift from LF diet to HF diets (*P* = 0.07, Fig. 3E). In dark phases cod/scallop fed mice tended to be more active than casein and chicken fed mice (*P* = 0.09, Fig. 3D) and a strong tendency towards higher total activity was seen in cod/scallop fed compared to casein and chicken fed mice (*P* = 0.06, Fig. 3E). Consistent with activity, EE was higher in dark than in light phases in LF fed mice. With the shift to HF diets, EE decreased in dark phases while no difference was seen between LF and HF feeding in light phases (Fig. 3F-G). No difference was seen between groups in light or dark phases while on LF diet, whereas EE tended to decrease (*P* = 0.08, Fig 3G) in casein fed compared to chicken and cod/scallop fed mice in light phases and increased in cod/scallop fed compared to casein fed mice during dark phases (Fig. 3G).

## Discussion

An increasing body of evidence supports a preventive role of HP diets against development of obesity. Less is known as to whether different protein sources consumed at normal dietary levels may differently affect energy balance. In the present study, we fed obesity-prone male C57BL/6J mice HF diets with either casein, chicken filet or a mixture of cod filet and scallop muscle as the protein source. Even though energy intake did not differ significantly, LF and chicken fed mice had higher feed efficiencies than the casein and cod/scallop fed mice, which after seven weeks of feeding translated into increased body masses and for the chicken fed mice also increased adipose tissue masses. Concomitantly, the chicken fed mice had deteriorated plasma lipid profile and enlarged liver mass with elevated hepatic TG levels. Thus, we demonstrate that different protein sources affect diet-induced obesity and associated co-morbidities in C57BL/6J mice when given at normal levels in a HF background diet.

Body fat accretion was reduced, evident as lower body mass gain, lower adipose tissue masses and reduced liver TG, in the casein and cod/scallop fed compared to the chicken fed mice. Interestingly, the apparent fat digestibility was reduced from an average of about 98% in LF, chicken and cod/scallop fed mice, to an average of about 95% in casein fed mice. Assuming that the apparent fat digestibility was constant for the entire seven week period, the casein fed mice absorbed approximately five g less fat than LF, chicken and cod/scallop fed mice. In mice, intake of a HF casein diet has previously been reported to cause higher fecal fat excretion and a leaner phenotype as compared to intake of a HF salmon diet [38]. Hence, it is likely that the reduced apparent fat absorption was a contributing factor to the reduced fat accretion in casein fed mice in the present study.

The cod/scallop fed mice maintained a lean phenotype, relative to chicken fed mice, without a reduction in fat absorption. To elucidate whether the protein sources modulated energy metabolism, we subjected the mice to indirect calorimetric measurements before onset of obesity at the transition from LF to HF feeding. HF diets have previously been shown to disturb feeding pattern and behavioral circadian rhythm in mice [39], such that the LF diet-induced fluctuations in RER between light and dark phases, reflecting different feed intake and substrate oxidations, are completely abolished after a switch to a casein based HF diet [40]. Accordingly, the RER was promptly reduced, and the differences in RER between light and dark phases disappeared after the switch to HF diets in the present study. There was no protein source effect on RER in the present study. However, following the transition from LF diet to HF diets EE decreased less in the cod/scallop fed than in casein fed mice, but we observed no significant difference in EE between chicken fed and cod/scallop fed mice that could explain the difference in adiposity. Our indirect calorimetry setup monitored gas exchange of each mouse for 1.9 minutes every 30 minutes, and it has been argued that the monitoring frequency has to be considerably higher in order to detect the 2–5% changes in diet-induced EE sufficient to elicit long term alterations on energy balance [41]. Decreased spontaneous locomotor activity has previously been demonstrated at the transition from LF to HF diets [39]. Accordingly, a decrease in spontaneous locomotor activity was observed in the light phases concurrent with a tendency towards decreased total activity (*P* = 0.07) after the shift from LF diet to HF diets in the present study. Importantly, cod/scallop feeding tended (*P* = 0.06) to avert this decrease in activity at the transition from LF to HF feeding. In line with this notion, we have previously observed an inverse correlation between locomotor activity and development of diet-induced obesity, without being able to detect differences in EE [21]. Indeed, whereas gas exchange was quantified at intervals (i.e. 1.9 min every 30 min), beam breaks were detected continuously, increasing the sensitivity of this measure as an indicator of EE. Therefore, differences in locomotor activity that nearly reached statistical significance (*P* = 0.06), are likely to reflect changes in EE that over time could explain the divergent fat accretion between the chicken and cod/scallop fed mice.

We have previously used another casein based HF diet (47 E% fat, 36 E% sucrose and 17 E% protein) to precipitate obesity and glucose intolerance in mice [12]–[14]. By increasing the fat content to 67 E% and reducing the sucrose content to 18 E%, the casein fed mice in the present study remained lean. Despite their lean phenotype, the casein fed mice were less glucose tolerant, when challenged in an O-GTT after six weeks of feeding. Cod protein intake has previously been associated with improved glucose metabolism in rats due to better peripheral insulin sensitivity as compared to casein feeding [30], [42], [43]. Moreover, in a randomized controlled intervention study with crossover design, insulin-resistant subjects exhibited improved insulin sensitivity [44] and reduced levels of the inflammatory marker high-sensitivity C-reactive protein after intake of a cod based relative to a meat and dairy based diet for four weeks [45]. Therefore, both in the present study, as well as in studies with rats and humans, intake of cod as compared to casein is associated with improved glucose metabolism.

During HF feeding, metabolic adaptations to the elevated fat load occur by increasing mitochondrial content and oxidative capacity in liver [46], [47] and skeletal muscle [40], [48]. As a strong regulatory interaction exists between lipid and carbohydrates oxidation [49], HF feeding represses the use of glucose as an energy substrate (i.e. glycolysis) [40], [46], a condition that could promote glucose intolerance. Based on the improved glucose clearance in the cod/scallop fed compared to casein fed mice in the present study as well as in HF cod fed rats reported by others [30], [43], it is evident that dietary protein source affects glucose metabolism. However, our data did not indicate higher glycolysis or glucose utilization in the cod/scallop fed as compared to the casein fed mice and further studies are needed to elucidate the underlying mechanisms for the differences in glucose clearance.

The present study was not designed to identify underlying mechanisms, merely to elucidate whether diets with casein, chicken filet or a mixture of cod filet and scallop muscle modulate diet-induced obesity. As locomotor activity can be stimulated [50], [51] and EE increased [52] by dietary taurine it is possible that the high taurine concentration of the cod/scallop diet contributed to the observed modulation of energy balance in the mice fed this diet. In addition, altered metabolism of branched-chain amino acid (BCAA) is likely associated with glucose dysregulation and the development of insulin-resistance [53]. In line with this notion, BCAA supplementation in a casein based HF diet impaired glucose tolerance in rats [54]. In the present study, the BCAA content was 39% higher in the casein diet than in the cod/scallop diet, which may have contributed to the observed differences in glucose tolerance. Elevated levels of BCAAs, leucine in particular, are associated with inhibition of insulin signaling through activation of the mammalian target of rapamycin pathway [55], [56]. The altered glucose metabolism observed in the present study could therefore be related to differences in leucine intake. However, arguing against this is the recent findings that leucine supplementation in drinking water diminished HF feeding induced insulin resistance in mice [57], [58]. Thus, further studies are needed to clarify whether varying amino acid content contributed to the observed differences in the present study.

In conclusion, protein sources are of importance for development of diet-induced obesity and associated co-morbidities in obesity-prone male C57Bl/6J mice fed HF diets. Whereas both casein and a mixture of cod and scallops prevented obesity, hepatic lipid accumulation and dyslipidemia, only cod/scallop fed mice remained glucose tolerant when challenged in an O-GTT. Further studies are needed to elucidate the underlying mechanisms for how the different protein sources induce these phenotypical alterations in HF fed mice.

## Supporting Information

Table S1 Genes and corresponding primer sequences used for qRT-PCR.
(PDF)
