# Supplementary material for: A Mixture of Cod and Scallop Protein Reduces Adiposity and Improves Glucose Tolerance in High-Fat Fed Male C57BL/6J Mice
Source: PLoS One. 2014 Nov 12;9(11):e112859. doi: 10.1371/journal.pone.0112859 (PMC4229262; doi:10.1371/journal.pone.0112859)
Supplement: Table S1 — Genes and corresponding primer sequences used for qRT-PCR. (PDF) [file pone.0112859.s001.pdf]

Supporting Information

Table S1. Genes and corresponding primer sequences used for qRT-PCR.

Abbreviation	Gene name	5'prime	3'prime
Srebf1	Sterol regulatory element-binding transcription factor 1	GGAGCCATGGATTGCACATT	GCTTCCAGAGAGGAGCCCAG
Acaca	Acetyl-Coenzyme A carboxylase alpha	TGCTGCCCCATCCCCGGG	TCGAACTCTCACTGACACG
Fasn	Fatty acid synthase	CTTCGCCAACTCTACCATGG	TTCCACACCCATGAGCGAGT
Scd-1	Stearoyl-CoA desaturase-1	GATGTTCCAGAGGAGGTACTACAAG	ATGAAGCACATCAGCAGGAGG
Dgat-1	Diacylglycerol acyltransferase-1	GGTGCCATCGTCTGCAAGA	CCACCAGGATGCCATACTTGA
Hmgcr	3-Hydroxy-3-Methylglutaryl-Coenzyme A reductase	ATCATCCTGACGATAACGCG	GCCAGCAATACCCAGAATGT
Pck-1	Phosphoenol pyruvate carboxykinase-1	CCACACCATTGCAATTATGC	CATATTTCTTCAGCTTGCGG
Hk2	Hexokinase 2	AGAGAACAAGGGCGAGGAG	GGAAGCGGACATCACAATC
Hk4	Hexokinase 4	CAAGAGGAGAGGGGACTTTG	TTGGCGGTCTTCATAGTAGC
Pfkl	Phosphofructokinase, liver, B-type	GCCTATCTCATCCAGCTACG	CTTGCTACTCAGGATTCGGT
Pklr	Pyruvate kinase liver and red blood cell	GAGTCTTCCCCTTGCTCTACC	CCTGTCACCACAATCACCA
Tbp	TATA-box binding proten	ACCCTTCACCAATGACTCCTATG	ATGATGACTGCAGCAAATCGC
